# TEMPURA: Database of Growth TEMPeratures of Usual and RAre Prokaryotes

**DOI:** 10.1264/jsme2.ME20074

**Published:** 2020-07-29

**Authors:** Yu Sato, Kenji Okano, Hiroyuki Kimura, Kohsuke Honda

**Affiliations:** 1 International Center for Biotechnology, Osaka University, 2–1 Yamada-oka, Suita, Osaka 565–0871, Japan; 2 Research Institute of Green Science and Technology, Shizuoka University, 836 Oya, Suruga-ku, Shizuoka 422–8529, Japan; 3 Department of Geosciences, Faculty of Science, Shizuoka University, 836 Oya, Suruga-ku, Shizuoka 422–8529, Japan

**Keywords:** growth temperature, database, prokaryote, genome, 16S ribosomal RNA

## Abstract

Growth temperature is one of the most representative biological parameters for characterizing living organisms. Prokaryotes have been isolated from various temperature environments and show wide diversity in their growth temperatures. We herein constructed a database of growth TEMPeratures of Usual and RAre prokaryotes (TEMPURA, http://togodb.org/db/tempura), which contains the minimum, optimum, and maximum growth temperatures of 8,639 prokaryotic strains. Growth temperature information is linked with taxonomy IDs, phylogenies, and genomic information. TEMPURA provides useful information to researchers working on biotechnological applications of extremophiles and their biomolecules as well as those performing fundamental studies on the physiological diversity of prokaryotes.

Living organisms adapt to changes in various environmental parameters, such as temperature, pH, pressure, and nutrient availability, in their natural habitats. Prokaryotes are often found in various extreme environments, ranging from arctic lakes (below 0°C) to deep-sea hydrothermal vents (higher than 100°C), and show wider diversity in their growth temperatures than eukaryotes (Tschitschko *et al.*, 2018; Dick, 2019). The growth temperatures of prokaryotes are important information not only for fundamental studies (*e.g.*, those on the mechanisms of temperature adaptation by living organisms and the exploration of limits of the biosphere), but also bioprospecting studies on commercially valuable thermotoleant enzymes and antifreeze proteins. However, the majority of studies have focused on a limited number of model extremophiles due to the time-consuming process of a literature search for rare thermophiles and psychrophiles (Haki and Rakshit, 2003).

Therefore, a comprehensive database of prokaryotic growth temperatures represents a promising tool for overcoming these limitations and facilitating both fundamental and biotechnological studies in relevant fields. A database summarizing the optimum growth temperatures of 1,072 prokaryotes has already been developed (Huang *et al.*, 2004), but is currently inaccessible (Modarres *et al.*, 2018). Although the optimum growth temperatures of 8,093 prokaryotes have been utilized to investigate the relationships between their growth temperatures and genome sequences, these data have not been published as a database (Sauer *et al.*, 2015, 2019). Therefore, we herein newly constructed a database of growth TEMPeratures of Usual and RAre prokaryotes (TEMPURA), which includes the minimum, optimum, and maximum growth temperatures of 8,639 prokaryotic strains. We also performed a correlation analysis between growths temperature and guanine-plus-cytosine (G+C) contents in prokaryotic DNA using data deposited in TEMPURA.

To construct the database, we manually collected the minimum (*T*_min_), optimum (*T*_opt_), and maximum (*T*_max_) growth temperatures of 8,639 strains (archaea, 549; bacteria, 8,090). Each strain deposited in TEMPURA has a direct link to the corresponding web page in the NCBI Taxonomy database (Sayers *et al.*, 2009, 2019). *T*_min_, *T*_opt_, and *T*_max_ of the strain was obtained from the oldest relevant study cited on the web page. When there was no relevant study in the Taxonomy database, the online version of Bergey’s Manual (https://onlinelibrary.wiley.com/doi/book/10.1002/9781118960608) was employed as a source of strain information. Strains without this set of growth temperature information were removed. When an optimum growth temperature was shown as a range, the average value was employed. Additionally, the lowest (Topt_low) and highest (Topt_high) values in the range were shown in TEMPURA. Based on growth temperatures, we divided strains into four groups as described previously (Madigan *et al.*, 2003; Moyer and Morita, 2007): psychrophiles and psychrotrophic microorganisms (*T*_opt_<20°C), mesophiles (20≤*T*_opt_<45°C), thermophiles (45≤*T*_opt_<80°C), and hyperthermophiles (80°C≤*T*_opt_).

TEMPURA was constructed on TogoDB (http://togodb.org), an online database that is freely accessed. It contains species names, taxonomy IDs, lineages (superkingdom, phylum, class, order, family, and genus), and genomic information (G+C content and genome size) in addition to growth temperatures. The lineage and genomic data of the isolated strains were obtained from the NCBI Taxonomy database and NCBI Genome database, respectively (Sayers *et al.*, 2009, 2019).

TEMPURA also includes information on various extremophiles: 209 psychrophiles and psychrotrophic microorganisms (3 archaea and 206 bacteria), 7,574 mesophiles (327 archaea and 7,247 bacteria), 731 thermophiles (104 archaea and 627 bacteria), and 125 hyperthermophiles (115 archaea and 10 bacteria). Among them, *Methanopyrus kandleri* strain 116 showed the highest *T*_max_ of 122°C (Takai *et al.*, 2008), whereas “Geogemma barossii” strain 121 and *Pyrolobus fumarii* strain 1A showed the highest *T*_opt_ of 106°C (Blöchl *et al.*, 1997; Kashefi and Lovley, 2003). These archaea were all isolated from deep-sea hydrothermal vents, which are one of the hottest places on Earth’s surface. On the other hand, the lowest *T*_min_ of –20°C was observed for *Planococcus faecalis* strain AJ003, a bacterium isolated from the stools of Antarctic penguins (Kim *et al.*, 2015), and the lowest *T*_opt_ was 2°C (or lower) for *Moritella profunda* strain 2674, a moderate-piezophilic bacterium isolated from Atlantic sediments (Xu *et al.*, 2003). Therefore, the strains that showed the highest *T*_max_ or lowest *T*_min_ were not necessarily the same as those with the highest or lowest *T*_opt_. TEMPURA also revealed the existence of rare strains growing in a wide temperature range. The largest difference between *T*_min_ and *T*_max_ was 60°C in *Kosmotoga olearia* strain TBF 19.5.1 (20 and 80°C), *Bacillus beveridgei* strain MLTeJB (5 and 65°C), and *Streptomyces thermoautotrophicus* strain UBT1 (10 and 70°C), which were isolated from a high-temperature oil field (DiPippo *et al.*, 2009), a highly alkaline and hypersaline lake (Baesman *et al.*, 2009), and the covering soil of a burning charcoal pile (Gadkari *et al.*, 1990), respectively. The large temperature gradient in an oil reservoir, diurnal temperature variations in a salt lake, and rapid heating by burning may had led to the adaptation of these strains to a wide range of temperatures.

TEMPURA is also useful for correlation analyses between growth temperatures and genomic information. In the present study, we performed correlation analyses between growth temperatures and G+C contents, which are related to the structural strengths of DNA and RNA or genome sizes as major nucleotide information (Fig. 1). No correlations were observed between the G+C content of a genomic sequence and *T*_min_, *T*_opt_, or *T*_max_ (R=–0.291 to –‍0.032) (Fig. 1a). On the other hand, strong positive correlations were found between the G+C contents of 16S ribosomal RNAs and growth temperatures in archaea (R=0.842 to 0.880) (Fig. 1b). Similar results were obtained in bacteria (R=0.378 to 0.404); however, correlation coefficients and symmetry in data distribution were lower than those in archaea. Higher coefficients (R=0.455 to 0.505) were observed when only thermophilic bacteria were subjected to the correlation analysis (Fig. S1), suggesting that a high G+C content, and thus, the structural strength of ribosomal RNAs are of importance for the thermal adaptation of prokaryotes, particularly at high temperatures. This result is consistent with previous findings showing higher G+C contents in the ribosomal RNAs of thermophiles and hyperthermophiles than in those of psychrophiles, psychrotrophic microorganisms, and mesophiles (Galtier and Lobry, 1997; Khachane *et al.*, 2005; Kimura *et al.*, 2006; Wang *et al.*, 2006; Kimura *et al.*, 2007, 2010, 2013). Data symmetry in thermophilic bacteria also appeared to be higher than that in the others (Fig. S1), which may be due to the presumably higher accuracy in the procedure to investigate the growth temperatures of these bacteria. The proportion of thermophilic and hyperthermophilic strains was significantly lower in bacteria (8.0%) than in archaea (40.1%). This may have resulted in the lower correlation coefficient in bacteria than in archaea. We also found that genome sizes showed a moderately negative correlation with growth temperatures in both archaea (R=–0.592 to –0.579) and bacteria (R=–0.378 to –0.313) (Fig. 1c). These results demonstrated that genomic DNA is slightly shorter in thermophiles and hyperthermophiles than in psychrophiles, psychrotrophic microorganisms, and mesophiles, which is consistent with previous findings (Sabath *et al.*, 2013). As described above, TEMPURA will be useful for obtaining and utilizing growth temperatures, which will accelerate fundamental and applicable studies in various fields.

## Citation

Sato, Y., Okano, K., Kimura, H., and Honda, K. (2020) TEMPURA: Database of Growth TEMPeratures of Usual and RAre Prokaryotes. *Microbes Environ ***35**: ME20074.

https://doi.org/10.1264/jsme2.ME20074

## Supplementary Material

Supplementary Material

## Figures and Tables

**Fig. 1.**
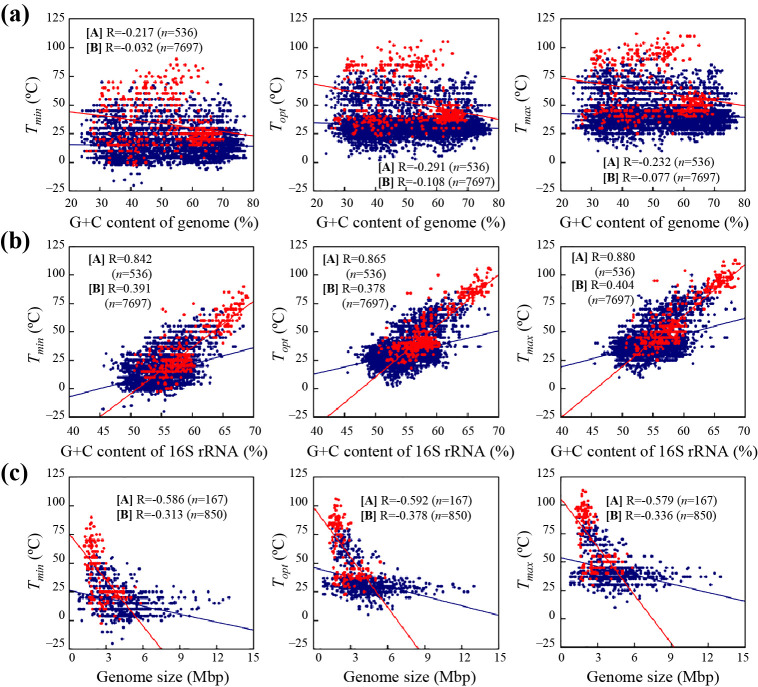
Correlations between genomic features and growth temperatures in archaeal ([A], red circle and red line) and bacterial strains ([B], blue circle and blue line). Each graph shows the correlation between (a) the G+C content of a complete genome, (b) the G+C content of 16S rRNA, and (c) the genome size with each growth temperature (*T*_min_, *T*_opt_, and *T*_max_). Correlations were examined using Pearson’s correlation coefficients (R values).
