# Supplementary material for: TEMPURA: Database of Growth TEMPeratures of Usual and RAre Prokaryotes
Source: Microbes Environ. 2020 Jul 29;35(3):ME20074. doi: 10.1264/jsme2.ME20074 (PMC7511790; doi:10.1264/jsme2.ME20074)
Supplement: Supplementary file 1 — Supplementary Material [file 35_20074_s1.pdf]

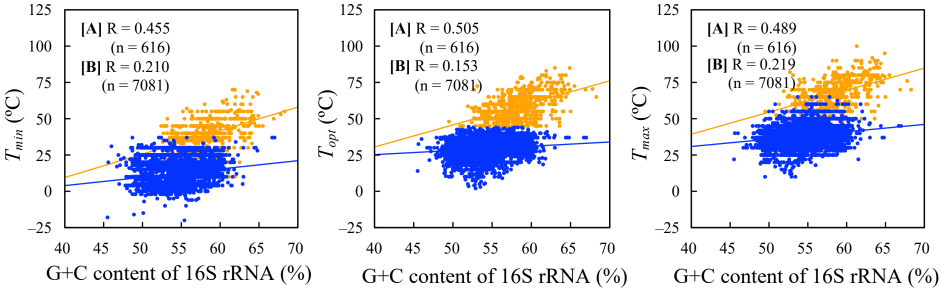

Fig. S1. Correlations between the G+C content of 16S rRNA and each growth temperature (T_{min}, T_{opt}, and T_{max}) in thermophilic and hyperthermophilic ([A], orange, and orange line), and the other bacterial strains ([B], blue circle, and blue line). Correlation was determined using the Pearson correlation coefficients (R values).
